# Correlation Between Dosimetric Parameters and Hematologic Toxicity in Cervical Cancer Patients Undergoing Intensity-Modulated Pelvic Radiotherapy

**DOI:** 10.3390/cancers18060992

**Published:** 2026-03-19

**Authors:** Shuang Zhao, Xi Yang, Lu Zhang, Duan Yang, Xuejiao Yang, Rui Wang, Shuangzheng Jia, Jusheng An, Manni Huang

**Affiliations:** Department of Gynecological Oncology, National Cancer Center/National Clinical Research Center for Cancer/Cancer Hospital, Chinese Academy of Medical Sciences and Peking Union Medical College, Beijing 100021, China; zs18846149423@163.com (S.Z.); yangxi_pumc@outlook.com (X.Y.); happyzl9003@163.com (L.Z.); yangduan1213@163.com (D.Y.); yangxj1206@163.com (X.Y.); wangrui.lucky0211@foxmail.com (R.W.); jiashuangzheng@cicams.ac.cn (S.J.)

**Keywords:** cervical cancer, hematologic toxicity, pelvic bone marrow, chemoradiotherapy, dosimetric parameters

## Abstract

**Objective:** This study aimed to elucidate the association between hematologic toxicity (HT) and pelvic bone marrow (PBM) dosimetric parameters in patients with cervical cancer (CC) undergoing radiotherapy (RT) combined with artificial intelligence (AI)-assisted organ at risk (OAR) delineation (Software Copyright Registration Number 2023SR0150365). Accurate delineation of bone marrow (BM) regions and analysis of radiation doses may provide a theoretical foundation for the application of AI in predicting HT. **Methods:** This retrospective study included 141 patients with CC who received chemotherapy (sequential or concurrent) and/or pelvic volumetric modulated arc therapy (VMAT) at the Department of Gynecology, Cancer Hospital of the Chinese Academy of Medical Sciences, between March 2019 and December 2019. PBM and its subregions (ilium, lower pelvis, lumbosacral spine, and femoral heads) were delineated using AI-based automatic segmentation of CT images. The volumes receiving 10–40 Gy (V10, V20, V30, V40) were calculated, and baseline clinical characteristics were assessed. HT endpoints included grade ≥ 2 (HT2+) and grade ≥ 3 (HT3+) leukopenia, neutropenia, anemia, or thrombocytopenia. Associations between dosimetric parameters and HT were evaluated using logistic regression models. **Results:** Of the 141 patients, 107 (75.8%) developed HT2+ and 33 (23.4%) developed HT3+. Univariate analysis showed that chemotherapy and age were correlated with HT2+. Multivariate analysis identified femoral head V30, femoral head V40, and chemotherapy as independent predictors of HT3+. **Conclusions:** This study highlights the potential of AI-based OAR delineation for assessing PBM dosimetric parameters in patients with CC. Optimizing RT to minimize BM dose and volume may mitigate HT and enhance treatment tolerance. In our cohort, receipt of combined neoadjuvant and concurrent chemotherapy (NACT+CCRT) was a stronger predictor of HT than most BM dosimetric parameters, suggesting that the systemic effect of chemotherapy may dominate the hematologic toxicity profile in this setting. Consequently, patients receiving this combined modality treatment are at particularly high risk for HT and warrant close hematologic monitoring.

## Simple Summary

This study investigated the relationship between radiation dose to the pelvic bone marrow and the development of hematologic toxicity (HT) in 141 patients with cervical cancer undergoing radiotherapy and chemotherapy. Using artificial intelligence to precisely delineate bone marrow regions, the researchers analyzed dose-volume parameters and clinical factors associated with HT. The results showed that 75.8% of patients developed grade ≥ 2 HT, and 23.4% developed grade ≥ 3 HT. Notably, patients receiving both neoadjuvant and concurrent chemotherapy had a threefold higher risk of severe HT compared to those receiving radiotherapy alone or with concurrent chemotherapy alone, making chemotherapy a stronger predictor of HT than most bone marrow radiation doses. Femoral head dose was also identified as an independent predictor. These findings highlight that the systemic effects of chemotherapy, rather than localized bone marrow irradiation, predominantly drive severe hematologic toxicity. This knowledge is valuable for clinical practice as it identifies a particularly high-risk patient population requiring close hematologic monitoring and suggests that modifying systemic therapy may be more effective than bone marrow dose constraints alone in mitigating treatment-related toxicity.

## 1. Introduction

CC is one of the most common and aggressive malignancies of the female reproductive system and represents a substantial global health burden. In 2022, approximately 662,044 new cases and 348,709 deaths were attributed to CC worldwide. China’s age-standardized incidence of CC (13.8 per 100,000) was slightly lower than the global average (14.1 per 100,000), and its mortality rate (4.5 per 100,000) was also lower than the global rate (7.1 per 100,000). Despite these favorable rates, China accounts for a substantial proportion of the global disease burden, with 150,659 new cases (22.8% of the world total) and 55,694 deaths (16.0%). China thus has the highest number of new CC cases and the second-highest number of deaths globally, after India [1]. CCRT, which combines RT and chemotherapy, is the standard treatment approach for locally advanced CC. A major adverse effect of CCRT is acute HT, which may manifest as increased risk of infection, higher treatment costs, reduced chemotherapy efficacy, and potential treatment interruptions or prolongation of the overall treatment duration. These complications can compromise therapeutic outcomes [2].

The European Society of Gynecological Oncology and the European Society for Radiotherapy and Oncology recommend IMRT techniques, including volume-modulated arc therapy (VMAT), for external RT in patients with CC [3]. IMRT optimizes dose distribution to the target volume while sparing organs at risk (OARs). Dosimetric studies have demonstrated that IMRT and VMAT reduce radiation doses to the bladder, rectum, and PBM without compromising dose distribution to the planned target volume [4,5]. Furthermore, optimized IMRT and VMAT protocols can reduce PBM dose without increasing dose exposure to surrounding OARs [6].

Determining a patient’s suitability for RT requires balancing anticipated treatment efficacy against potential toxicity. BM is particularly vulnerable to radiation because it contains hematopoietic stem cells, which are among the most radiosensitive cell types in the body. In adults, active hematopoietic BM is located in the pelvis, accounting for approximately 34.5% of total active marrow, followed by 16.6% in the lumbar vertebrae and 4.5% in the proximal femoral heads [7]. Over the past two decades, numerous studies have investigated BM protection strategies during pelvic RT, leading to proposed dose constraints and clinical guidelines. The National Comprehensive Cancer Network guidelines for radical RT in CC recommended BM constraints include V10 < 80% and V20 < 66% (soft constraints) and V10 < 90% and V20 < 70% (hard constraints). For postoperative adjuvant RT, soft and hard limits are V10 < 10% and V20 < 30%, respectively [8]. However, achieving an optimal balance between HT mitigation and antitumor efficacy while adhering to these dose limitations remains a clinical challenge.

Therefore, this study retrospectively analyzed dosimetric parameters and associated clinical data in patients with CC undergoing RT and chemotherapy to identify specific factors influencing HT.

## 2. Materials and Methods

### 2.1. Case Selection

This retrospective analysis included 141 patients with CC who underwent preoperative neoadjuvant, radical, or postoperative adjuvant RT at the Cancer Hospital of the Chinese Academy of Medical Sciences (National Cancer Center) between March 2019 and December 2019. The study was approved by the Institutional Review Board of the Institute.

### 2.2. Treatment Methods

#### 2.2.1. CCRT

In total, 108 patients (83.7%) received concurrent RT and were scheduled for weekly platinum-based sensitizing chemotherapy with concurrent external beam RT (EBRT), provided the following hematologic criteria were met: absolute neutrophil count > 1.0 × 10^9^/L, platelet count > 50 × 10^9^/L, hemoglobin > 75 g/L, white blood cell count > 3.0 × 10^9^/L, and creatinine clearance > 50 mL/min. During treatment, most patients received up to five cycles of platinum-based chemotherapy.

#### 2.2.2. Radiotherapy

Patients were positioned supine for computed tomography (CT) scanning. CT images with 5 mm slice thickness from above the diaphragm to 2 cm below the perineum were transferred to the PINNACLE planning system (Philips Radiation Oncology Systems, Milpitas, CA, USA). All visible gross lesions identified on CT and magnetic resonance imaging (MRI), including metastatic pelvic lymph nodes and the primary cervical tumor, were defined as the gross tumor volume (GTV). The clinical target volume (CTV) included the GTV and areas at high risk of harboring microscopic disease, encompassing the cervix, parametria, vagina, uterine corpus, and lymph node drainage regions (e.g., common, internal, and external iliac, obturator, and presacral nodes). The PTV was created by adding a 5 mm margin to the CTV in the left–right, anterior–posterior, and superior–inferior axes. OARs included the bladder, femoral heads, rectum, sigmoid colon, small bowel, spinal cord, and pelvic bones. The prescribed dose to the PTV was 45–50.4 Gy. Doses to OARs were kept as low as reasonably achievable, adhering to the following constraints: bladder V50 < 20% and V40 < 50%; femoral heads V50 < 5% and V30 < 30%; small bowel and colon V40 < 50%, Dmax < 52 Gy; sigmoid colon V40 < 60%, Dmax < 52 Gy; rectum V50 < 20%, V40 < 50%; spinal cord Dmax < 40 Gy; and pelvic bones V30 < 50%. The planning objective was to deliver 95% of the prescribed dose to at least 95% of the PTV while respecting OAR dose limits.

### 2.3. Pelvic Delineation and Dose Parameter Selection

**Figure 1:**
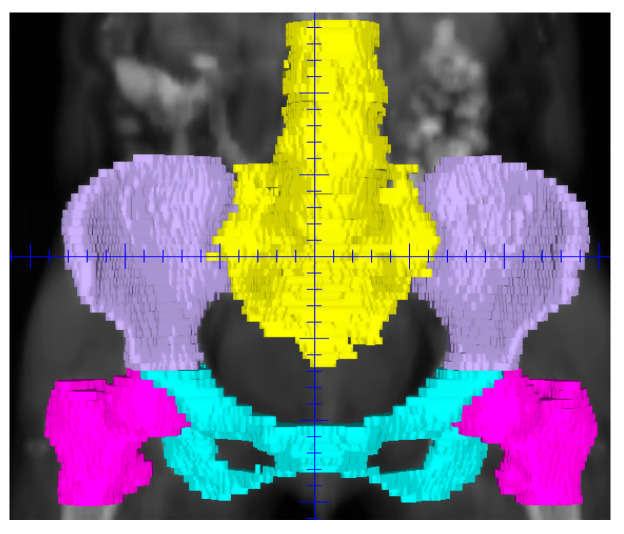
AI-based segmentation of pelvic bone marrow subregions.

PBM was defined as the external contours of all pelvic bones and subdivided into four regions: ilium, lower pelvis, lumbosacral spine, and femoral heads (Figure 1). OARs were delineated using AI-based auto-segmentation software (copyright registration number 2023SR0150365). All AI-generated contours were reviewed and, if necessary, manually edited by a senior radiation oncologist to ensure anatomical accuracy before dose calculation. Dose–volume histograms were used to extract mean dose and V10, V20, V30, and V40 for PBM and each subregion, where Vx represents the percentage volume receiving ≥x Gy.

### 2.4. Hematologic Toxicity Assessment

All patients underwent baseline complete blood count (CBC) assessment and weekly CBC monitoring during treatment. Parameters monitored included hemoglobin, total white blood cell count, absolute neutrophil count, and platelet count. Adverse events were graded according to the Common Terminology Criteria for Adverse Events version 4.0, with grade ≥ 2 HT (HT2+) and grade ≥ 3 HT (HT3+) considered events. Pelvic brachytherapy dose was not considered, as the last hematologic event was recorded before brachytherapy. The incidence and severity (grades 1–4) of HT were recorded for each blood cell lineage.

### 2.5. Statistical Methods

Continuous variables included body mass index (BMI), age, and dosimetric parameters. Univariate logistic regression analysis was performed to assess associations between clinical and dosimetric parameters with HT2+ and HT3+. Variables with *p* < 0.10 in univariate analysis were entered into multivariate logistic regression models to identify independent predictors of HT. All statistical tests were two-sided, and *p* < 0.05 was considered statistically significant.

## 3. Results

### 3.1. Patient Characteristics

Table 1 summarizes the baseline clinical characteristics of the 141 patients. Median age at diagnosis was 54 years (range, 25–84 years). The majority of patients had clinical stage III disease (47.5%). Most patients (62.4%) had a BMI < 24 kg/m^2^. Median RT duration was 41 days. Concurrent platinum-based chemotherapy was administered to 118 patients (83.7%), and 35 patients (24.8%) received neoadjuvant chemotherapy (NACT).

### 3.2. Bone Marrow Dosimetric Parameters

Dosimetric parameters for PBM and its subregions are presented in Table 2. The median mean dose to PBM was 27.9 Gy.

### 3.3. Hematologic Toxicity

During treatment, 134 of 141 patients (95.0%) developed leukopenia. The incidences of acute neutropenia, thrombocytopenia, and anemia were 68.8%, 24.1%, and 53.2%, respectively. Grade ≥ 2 leukopenia, neutropenia, anemia, and thrombocytopenia occurred in 70.9%, 36.9%, 16.3%, and 12.0% of patients, respectively (Table 3).

Of the 141 patients, 107 (75.9%) experienced HT2+ during chemoradiotherapy. Among these, 100 patients (93.4%) had grade ≥ 2 leukopenia and/or neutropenia, 12 (11.2%) had concurrent grade ≥ 2 leukopenia/neutropenia and anemia, 12 (11.2%) had concurrent grade ≥ 2 leukopenia/neutropenia and thrombocytopenia, and 7 (6.5%) had involvement of all three lineages (Table 4).

### 3.4. Predictive Indicators of Hematologic Toxicity

Univariate analysis identified age and chemotherapy as factors associated with HT2+ (Table 5). Patients who received both NACT and CCRT had a higher probability of developing HT2+. In multivariate analysis, femoral head V30, femoral head V40, and chemotherapy remained independent predictors of HT3+ (Table 6).

## 4. Discussion

Acute hematologic toxicity (HT) is a common adverse event in patients undergoing sequential or concurrent chemoradiotherapy (CCRT). Furthermore, acute HT may delay or prevent the delivery of chemotherapy or radiotherapy (RT), thereby adversely affecting patient prognosis. Han et al. and Duenas-Gonzalez et al. observed that 40–60% cervical cancer (CC) and rectal cancer patients who received CCT experienced grade ≥ 3 myelosuppression [9,10]. Therefore, it is crucial to reduce acute HT incidence in patients with cancer undergoing RT.

Different studies have investigated the dosimetric effects of pelvic bone marrow (PBM) in patients receiving RT; however, a comprehensive analysis of risk factors associated with grade ≥ 2 HT remains elusive and warrants further investigation in clinical radiation oncology. This study investigated the relationship between artificial intelligence (AI)-delineated PBM dosimetric parameters and acute HT in 141 patients with CC receiving RT and chemotherapy. In our cohort, the incidences of grades 1, 2, 3, and 4 HT were 20.6% (*n* = 29), 52.4% (*n* = 74), 22.6% (*n* = 32), and 0.7% (*n* = 1), respectively. The proportions of patients developing grade ≥ 2 HT (HT2+) and ≥3 HT (HT3+) were 75.8% and 23.4%, respectively. The HT3+ rate of 23.4% is consistent with the 20–25% reported in the RTOG 0418 study of postoperative intensity-modulated RT (IMRT) for gynecologic cancer [11]. However, the incidence of HT2+ in our cohort was higher than that reported in the RTOG 0418 study. This may be explained by the inclusion of patients who received neoadjuvant chemotherapy (NACT) before RT. Notably, some patients with a large primary tumor burden underwent NACT to reduce tumor volume and facilitate surgical resection. In contrast, others already exhibited evidence of bone marrow suppression even before CCRT initiation. Collectively, these factors may have contributed to the increased risk of severe chemotherapy-related HT observed in this cohort.

Several studies have investigated the association between clinical factors and HT in patients with CC receiving RT, with largely negative findings. Huang et al. found that myelosuppression was not associated with clinical stage, age, surgery, RT, chemotherapy cycles, or RT technique in 155 patients [12]. Similarly, Niu et al. found no correlation among myelosuppression and comorbidities, clinical stage, tumor classification, age, chemotherapy regimen, or number of uterine artery infusion chemotherapy courses [13]. Kumar et al. demonstrated that factors such as chemotherapy regimen (cisplatin vs. carboplatin), age, performance status, number of chemotherapy cycles, BMI, and para-aortic irradiation were not predictive of HT [14]. In contrast, a study identified that grade ≥ 2 leukopenia was associated with the number of chemotherapy cycles, while grade ≥ 2 neutropenia was associated with tumor stage and chemotherapy cycles [15]. In the present study, univariate analysis revealed no significant associations between acute myelosuppression and BMI, number of chemotherapy cycles, or clinical stage, which is consistent with the majority of past reports.

The most notable finding of this study is that patients receiving NACT and CCRT had a substantially higher risk of HT3+ (OR = 3.08) than those receiving CCRT or RT alone. This suggests that systemic chemotherapy, rather than bone marrow (BM) irradiation dose, is the primary driver of severe HT in this setting. This observation aligns with Manus et al., who identified concurrent chemotherapy as the strongest predictor of treatment interruptions (OR = 42.1) [16]. Chen et al. highlighted that neutrophil decline is affected by the number of CCT cycles and the BM irradiation dose, although chemotherapy may be more important [17]. Similarly, Cheng et al. found that more potent chemotherapy regimens (FOLFOX) obscured the dose–effect relationship observed with milder regimens (5-FU) [18]. A plausible explanation is that NACT compromises BM reserve before RT, rendering patients more susceptible to subsequent radiation-induced injury. This finding has important clinical implications: patients receiving NACT plus CCRT warrant close hematologic monitoring, and modifying systemic therapy may be more effective compared to BM dose constraints alone in mitigating HT.

Multiple studies have reported significant associations between PBM dose parameters and HT [6,19,20,21,22,23,24,25,26,27]. Konnerth et al. summarized PBM dose thresholds from 2006 to 2024: V10 < 90–95%, V20 < 65–86.6%, and V40 < 22.8–40% for grade ≥ 2 HT [25]. Similar associations have been observed in patients with rectal cancer [23,26] and validated using functional imaging modalities such as single-photon emission computed tomography (SPECT) [28]. In the RTOG 0418 trial, maintaining V40 < 37% reduced the incidence of grade ≥ 2 HT from 75% to 40%, whereas V10–V20 showed no similar predictive value [11]. Additionally, high-dose irradiation of large BM volumes (PBM-V40 ≥ 40% or PBM-V50 ≥ 15%) has been associated with increased neutropenia risk [29].

In addition to these volumetric parameters, the present study identified femoral head dose as an independent predictor of HT3+, a finding that warrants further discussion. Although the proximal femur contains only 4.5% of active BM [7], its consistent irradiation in pelvic fields may contribute to cumulative stem cell damage. However, the paradoxical finding that femoral head V30 was associated with increased risk of HT3+ (OR = 1.231, *p* = 0.013) while V40 appeared protective (OR = 0.460, *p* = 0.020) warrants careful interpretation.

A strong correlation was observed between femoral heads V30 and V40, indicating substantial multicollinearity. When highly correlated variables are included in the same regression model, coefficient estimates may become unstable and can even reverse direction [30]. This statistical artifact likely explains the opposing effect estimates. In addition, the clinical volume receiving ≥40 Gy to the femoral heads was extremely small in our cohort (median V40 = 0.2%, 0–7.9%). Among the few patients with measurable V40 values, the limited volume of high-dose irradiation may not be biologically meaningful, and the apparent protective effect likely represents a statistical anomaly rather than a true biological phenomenon. Furthermore, given the relatively small number of HT3+ events (*n* = 33) relative to the number of predictors included in the multivariate model, these estimates should be viewed as hypothesis-generating rather than definitive. Future studies with larger sample sizes and functional BM imaging are needed to validate these findings. Despite these statistical considerations, the overarching clinical implication remains clear: minimizing the volume of femoral heads receiving high-dose radiation (≥30 Gy) may help reduce the risk of severe HT.

In addition to the dosimetric findings, the methodological aspects of this study warrant further discussion. AI-based auto-segmentation has emerged as a promising tool to improve efficiency and consistency in radiation oncology [31,32,33]. In this study, we used AI-assisted contouring to delineate PBM and its subregions to standardize the delineation process and reduce interobserver variability, a major source of inconsistency in dosimetric studies. All AI-generated contours were meticulously reviewed and, where necessary, manually adjusted by a senior radiation oncologist to ensure anatomical accuracy before dose calculation. However, this study did not evaluate AI performance metrics (e.g., Dice similarity coefficient) or compare AI-generated contours with manual contours. AI served solely to ensure consistent delineation across patients. Future studies specifically designed to validate AI-based PBM segmentation algorithms are needed to establish their clinical utility. A primary strength of this study is the use of AI to standardize contouring across a relatively large cohort of 141 patients, thereby reducing a major source of variability in dosimetric analyses. Additionally, the evaluation of less-studied subregions, such as the femoral heads, provides novel insights.

## 5. Limitations

Our study has several limitations. First, its retrospective design introduces potential selection bias. Second, although AI-assisted contouring was applied, functional BM imaging, such as SPECT or MRI, was not used; therefore, the spatial distribution of active BM could not be assessed. Future studies should incorporate functional imaging to optimize dose constraints. Third, nutritional status, a known confounder for HT, was not assessed. Fourth, with only 33 HT3+ events, the multivariate model may be underpowered, and the findings should be confirmed in larger prospective cohorts. Fifth, heterogeneity in chemotherapy regimens (cisplatin vs. carboplatin and number of cycles) could not be fully adjusted because of sample size limitations. Finally, some patients received NACT before RT. Although complete blood counts were obtained before RT, prior NACT may correlate with increased HT during CCRT or RT.

## 6. Conclusions

In summary, this study demonstrates that among patients with CC receiving RT and chemotherapy, receipt of NACT plus CCRT is a stronger predictor of HT than most BM dosimetric parameters. This suggests that the systemic effects of chemotherapy dominate the HT profile in this setting. Consequently, patients receiving this combined modality treatment are at particularly high risk for HT and require close hematologic monitoring. Although the opposing effect estimates observed for femoral head V30 and V40 are likely attributable to statistical multicollinearity rather than a true biological phenomenon, the clinical recommendation to minimize femoral head irradiation (≥30 Gy) is valid. Prospective studies incorporating functional BM imaging are needed to establish more precise and individualized dose constraints.

## Figures and Tables

**Table 1:** The clinical baseline characteristics of cervical cancer patients.

Characteristics	*n*
Number of patients (%)	141 (100)
Median age [range]	54 (25–84)
FIGO (%)	
I	24 (17)
II	39 (27.7)
III	67 (47.5)
IV	5 (3.5)
NA/M/R	6 (4.3)
BMI	
<24	88 (62.4)
≥24	53 (37.6)
Median prescription dose to PTV (Gy, range)	50 (45–50.4)
Prescription dose to SIB (Gy, median, range)	5 (0–15)
Initial treatment	
NACT (YES)	35 (24.8)
NACT (NO)	106 (75.2)
CCRT (YES)	118 (83.7)
CCRT (NO)	23 (16.3)
CCT cycles	
≤3	54 (38.3)
>3	87 (61.7)
RT days	
Median (range)	41 (32–89)

Abbreviations: BMI: body mass index; FIGO: International Federation of Gynecology and Obstetrics; NACT: Neoadjuvant Chemotherapy; CCRT: Concurrent Chemoradiotherapy; CCT: Concurrent Chemotherapy; NA: Not Available; M: Metastasis; R: Recurrence; PTV: Planned target volume.

**Table 2:** Dosimetric parameters of pelvic bone marrow and subregion.

Parameter	Median Value (Range)
PBM	
Mean dose (cGy)	2791.6 (825.1–3393)
V10 (%)	98.04 (71.92–100)
V20 (%)	65.62 (40.67–90.62)
V30 (%)	40.59 (30.04–59.9)
V40 (%)	21.54 (12.99–37.92)
Femoral heads	
Mean dose (cGy)	1352 (909.1–2217)
V10 (%)	60.33 (34.30–98.52)
V20 (%)	18.68 (3.93–63.81)
V30 (%)	5.5 (0.62–21.77)
V40 (%)	0.24 (0–7.94)
Ilium	
Mean dose (cGy)	2630.2 (770.1–3140.1)
V10 (%)	99.47 (9.61–100)
V20 (%)	59.34 (35.67–88.71)
V30 (%)	31.88 (18.67–56.66)
V40 (%)	15.83 (4.93–30)
Lower pelvic	
Mean dose (cGy)	3075.8 (2638.7–3918.7)
V10 (%)	96.49 (87.35–100)
V20 (%)	74.03 (56.02–100)
V30 (%)	51.62 (37.48–92.86)
V40 (%)	30.79 (19.21–52.16)
Lumbosacral Spine	
Mean dose (cGy)	3590.3 (349.6–4089.3)
V10 (%)	98.57 (91.21–100)
V20 (%)	89.55 (74.35–97.94)
V30 (%)	73.18 (55.34–94.77)
V40 (%)	41.71 (28.13–64.21)

Abbreviations: V10, V20, V30, V40: volume receiving 10, 20, 30, 40 Gy or more; PBM: Pelvic Bone Marrow.

**Table 3:** The incidence of acute hematologic toxicity.

Toxicity	Grade (%)				
	0	1	2	3	4
WBC	7 (5.0)	34 (24.1)	74 (52.5)	26 (18.4)	0 (0.0)
HB	66 (46.8)	52 (36.9)	16 (11.3)	7 (5.0)	0 (0.0)
PLT	107 (75.9)	17 (12.1)	14 (9.9)	3 (2.1)	0 (0.0)
NEUT	44 (31.2)	44 (31.2)	43 (30.5)	9 (6.4)	1 (0.7)

Abbreviations: WBC: White Blood Cell Count; HB: Hemoglobin; PLT: Platelets; NEUT: Neutrophil.

**Table 4:** The proportion of patients with HT2+ during radiotherapy.

Characteristic	*n* (%)
Leukopenia and/or neutropenia	100 (93.4%)
Leukopenia and/or neutropenia + anemia	12 (11.2%)
Leukopenia and/or neutropenia + thrombocytopenia	12 (11.2%)
Involvement of all three lineages	7 (6.5%)
Total HT2+ patients	107 (100%)

Abbreviations: Data are presented as number of patients (%). HT2+: Hematologic toxicity of grade ≥ 2.

**Table 5:** Univariate analysis of acute hematologic toxicity by dosimetric parameters, clinical features, and treatment pattern.

Parameter	*p*-Value	Odds Ratio (95% CI)	*p*-Value	Odds Ratio (95% CI)
	HT2+		HT3+	
Age	0.009	0.956 (0.925–0.989)	0.331	0.985 (0.954–1.016)
BMI	0.838	0.988 (0.881–1.108)	0.296	0.935 (0.825–1.060)
Pelvic Bone				
Dmean	0.464	1.001 (0.998–1.003)	0.601	1.001 (0.998–1.003)
V10	0.881	1.0068 (0.912–1.113)	0.669	1.025 (0.916–1.147)
V20	0.793	0.993 (0.946–1.043)	0.761	0.992 (0.943–1.044)
V30	0.364	1.032 (0.964–1.104)	0.744	1.011 (0.948–1.078)
V40	0.290	1.056 (0.955–1.168)	0.375	1.044 (0.950–1.147)
Femoral heads				
Dmean	0.883	1.000 (0.998–1.001)	0.979	1.000 (0.998–1.002)
V10	0.585	0.992 (0.965–1.020)	0.439	0.989 (0.961–1.018)
V20	0.730	1.007 (0.968–1.047)	0.372	1.01 (0.980–1.056)
V30	0.991	0.999 (0.910–1.097)	0.426	1.038 (0.947–1.137)
V40	0.282	0.872 (0.680–1.119)	0.435	0.875 (0.626–1.223)
Ilium				
Dmean	0.140	1.001 (1.000–1.003)	0.409	1.001 (0.999–1.003)
V10	0.533	0.997 (0.910–1.050)	0.509	1.027 (0.950–1.109)
V20	0.557	0.988 (0.950–1.028)	0.737	0.993 (0.953–1.035)
V30	0.369	1.027 (0.970–1.087)	0.838	1.006 (0.952–1.063)
V40	0.222	1.064 (0.963–1.175)	0.278	1.052 (0.960–1.153)
Lower pelvic				
Dmean	0.775	1.000 (0.998–1.001)	0.603	1.000 (0.998–1.001)
V10	0.652	1.028 (0.912–1.160)	0.661	0.973 (0.862–1.099)
V20	0.463	0.984 (0.944–1.026)	0.518	0.986 (0.944–1.030)
V30	0.802	0.994 (0.952–1.039)	0.477	0.983 (0.938–1.030)
V40	0.707	0.987 (0.923–1.055)	0.506	0.976 (0.910–1.048)
Lumbosacral Spine				
Dmean	0.551	1.000 (0.998–1.001)	0.551	1.000 (0.998–1.001)
V10	0.392	0.882 (0.662–1.176)	0.392	0.882 (0.662–1.176)
V20	0.894	0.995 (0.930–1.065)	0.894	0.995 (0.930–1.065)
V30	0.802	0.994 (0.952–1.039)	0.356	0.979 (0.937–1.024)
V40	0.681	0.989 (0.938–1.043)	0.681	0.989 (0.938–1.043)
Chemotherapy (NACT/CCRT)	0.003	3.042 (1.442–6.414)	0.006	2.79 (1.346–5.793)

Abbreviations: Dmean: Mean Dose.

**Table 6:** Multivariate analysis of clinical features and dosimetric parameters.

Parameter	*p*-Value	Odds Ratio (95%CI)	*p*-Value	Odds Ratio (95%CI)
	HT2+		HT3+	
Age	0.167	1.002 (1.000–1.004)	-	-
Femoral heads	-	-	-	-
V30	-	-	0.013	1.231 (1.045–1.450)
V40	0.167	0.830 (0.638–1.081)	0.020	0.460 (0.239–0.885)
ilium	-	-	-	-
Dmean	0.067	1.002 (1.000–1.004)	-	-
V30	-	-	0.078	0.899 (0.798–1.012)
V40	-	-	0.069	1.211 (0.985–1.487)
Chemotherapy (NACT/CCRT)	0.050	2.394 (1.000–5.733)	0.006	3.075 (1.372–6.891)

Note: A strong correlation was observed between femoral head V30 and V40, indicating substantial multicollinearity. When highly correlated variables are entered into the same regression model, coefficient estimates can become unstable and may reverse direction. Therefore, the opposing directions of effect for V30 (OR > 1, indicating increased risk) and V40 (OR < 1, suggesting a protective effect) should be interpreted as a statistical artifact rather than a true biological phenomenon.

## Data Availability

The data that support the findings of this study are available from the corresponding authors, M.H. and J.A., upon reasonable request.
